# The Relationship between Dental Fear and Anxiety, General Anxiety/Fear, Sensory Over-Responsivity, and Oral Health Behaviors and Outcomes: A Conceptual Model

**DOI:** 10.3390/ijerph19042380

**Published:** 2022-02-18

**Authors:** Leah I. Stein Duker, Mollianne Grager, Willa Giffin, Natasha Hikita, José C. Polido

**Affiliations:** 1 Mrs. T.H. Chan Division of Occupational Science and Occupational Therapy, Herman Ostrow School of Dentistry, University of Southern California, Los Angeles, CA 90089, USA; wgiffin@usc.edu (W.G.); nhikita@usc.edu (N.H.); 2 Leaps and Bounds of Washington, Seattle, WA 98136, USA; mollygrager@gmail.com; 3 Children’s Hospital Los Angeles, Los Angeles, CA 90027, USA; jpolido@chla.usc.edu

**Keywords:** dental anxiety, dental fear, oral health behaviors, general anxiety, sensory over-responsivity, dental behavior management problems

## Abstract

Dental fear and anxiety (DFA) is common across the lifespan and represents a barrier to proper oral health behaviors and outcomes. The aim of this study is to present a conceptual model of the relationships between DFA, general anxiety/fear, sensory over-responsivity (SOR), and/or oral health behaviors and outcomes. Two rounds of literature searches were performed using the PubMed database. Included articles examined DFA, general anxiety/fear, SOR, catastrophizing, and/or oral health behaviors and outcomes in typically developing populations across the lifespan. The relationships between the constructs were recorded and organized into a conceptual model. A total of 188 articles were included. The results provided supporting evidence for relationships between DFA and all other constructs included in the model (general anxiety/fear, SOR, poor oral health, irregular dental attendance, dental behavior management problems [DBMP], and need for treatment with pharmacological methods). Additionally, SOR was associated with general anxiety/fear and DBMP; general anxiety/fear was linked to poor oral health, irregular attendance, and DBMP. This model provides a comprehensive view of the relationships between person factors (e.g., general anxiety/fear, SOR, and DFA) and oral health behaviors and outcomes. This is valuable in order to highlight connections between constructs that may be targeted in the development of new interventions to improve oral health behaviors and outcomes as well as the experience of DFA.

## 1. Introduction

Dental fear is defined as a negative reaction to specific threatening stimuli associated with dental treatment, whereas dental anxiety is an excessive or impairing negative emotional state experienced by dental patients [1,2]. These terms are often used interchangeably or even combined as a single term, *dental fear and anxiety* (DFA) [1,2], which has been linked to many negative oral health behaviors and outcomes (e.g., [3,4,5,6,7,8,9,10,11]). DFA is primarily associated with previous negative dental experiences and most often develops in childhood [12,13,14,15,16]; however, literature suggests that DFA is common across the lifespan. Pediatric prevalence estimates range from 5% to 42%, with most reports clustering around 20% [2,5,16,17,18]. Similarly, 11–32% of adults report experiencing DFA [16,19,20,21,22].

A number of reviews have focused on DFA, including examinations of prevalence [2,16,17,22,23,24], trajectory over the lifespan [16], DFA-specific assessments [1,25,26], and management techniques [16,24,27,28]. Likewise, much research has reported—with overall inconsistent findings—on the relationship between DFA and sociodemographic characteristics, including age, gender, education, race/ethnicity, geographic location, and culture [2,29,30,31,32,33,34,35,36]. However, less attention has been paid to the relationship between DFA and other person factors such as general anxiety/fear and over-responsivity to sensory stimuli. Similar to DFA, general anxiety/fear and sensory over-responsivity (SOR) have been linked to negative oral health behaviors and outcomes in typical and clinical populations (e.g., [37,38,39,40,41,42,43,44]). Therefore, the purpose of this paper is to synthesize the current literature in order to present a conceptual model of DFA, other person factors which have the potential to be targeted in future interventions (e.g., general anxiety/fear and sensory over-responsivity), and their associations with each other and oral health behaviors and outcomes.

## 2. Materials and Methods

### 2.1. Search Strategy

A literature search was performed in an iterative manner, using the PubMed database, with the aim of identifying articles that examined DFA, other person factors, and their relationship with oral health behaviors and outcomes. Initial search terms included: dental fear, dental anxiety, dental behavior, oral health, caries, sensory processing, sensory over-responsiv*, sensory sens*, and combinations of these terms. Based on abstract review, the relevant publications were selected and reviewed. Following the review of these articles, new search terms were incorporated, such as: general anxiety, anxiety, catastrophizing, sedation, general anesthesia, dental attendance, tooth decay, dental behavior management problems, and oral health-related quality of life. Reference lists from relevant literature (including systematic reviews and meta-analyses) were reviewed to identify additional articles. Related articles and “cited by” queries in PubMed and Google Scholar databases were also reviewed for all articles included.

### 2.2. Inclusion and Exclusion Criteria

Articles were included if they examined the relationship between person factors (e.g., DFA, SOR, general anxiety/fear, and catastrophizing) and oral health behaviors and outcomes across the lifespan and were published in English. Systematic reviews, meta-analyses, unpublished data, and studies purposively recruiting clinical populations were excluded. As this study aimed to examine person factors that influence oral health behaviors and outcomes that may be targeted in future interventions, other articles focusing on constructs such as age, gender, race/ethnicity, parental DFA, socioeconomic status, and dentist behavior were excluded.

### 2.3. Model Development

**Figure 1:**
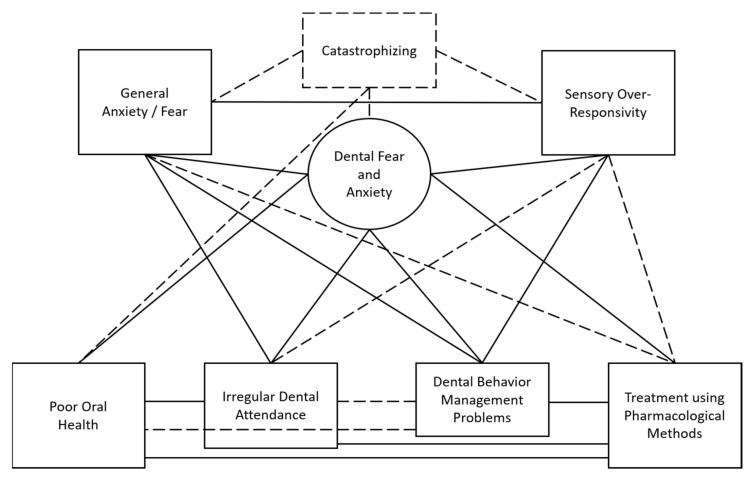
Preliminary conceptual model. Note. Dashed lines indicate relationships with support from only two or fewer independent studies (e.g., did *not* meet required inclusion criteria for final model).

**Figure 2:**
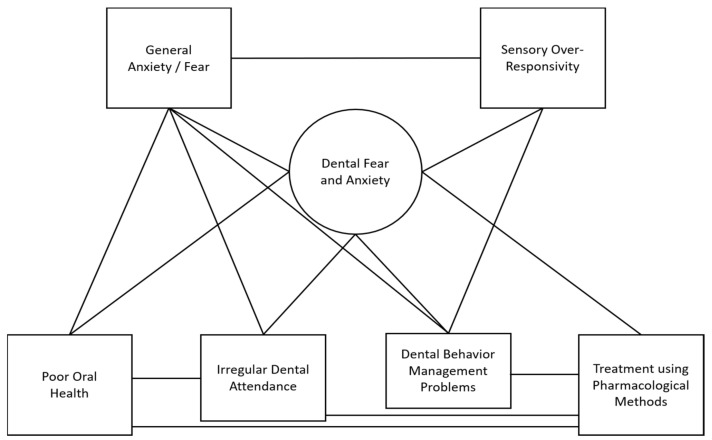
Conceptual model of the relationships between dental fear and anxiety, general anxiety/fear, sensory over-responsivity, and oral health behaviors and outcomes.

Following a review of articles from the initial search, several constructs related to DFA and/or SOR were identified by the authors (e.g., irregular attendance, oral health-related quality of life, and dental behavior management problems). In the second round of literature searches, articles examining these constructs and other person factors (general anxiety/fear and catastrophizing) were identified. Articles that met the inclusion criteria were reviewed and relationships between constructs recorded. In an iterative process, constructs were organized into a conceptual model to visually depict the associations between factors. As 97% of the included studies did not examine a causal link between constructs, lines were used in lieu of arrows so as not to infer relationship directionality (see Figure 1 for preliminary conceptual model). In the final model, lines were included only if three or more independent studies reported relationships between constructs (see Figure 2 for final conceptual model).

## 3. Results

**Figure 3:**
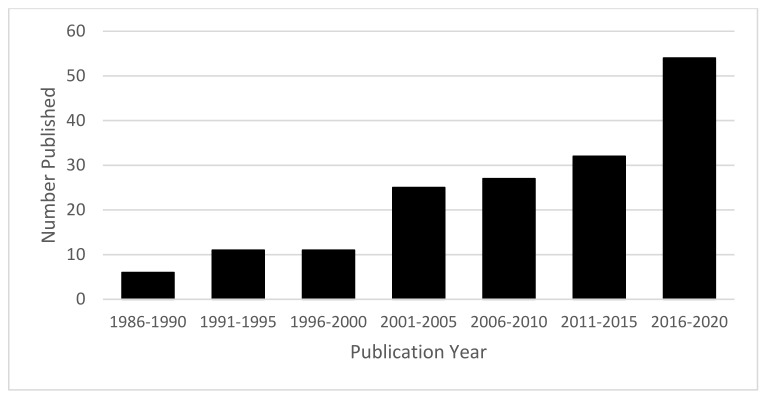
Number of articles included in conceptual model by year of publication. Note. Articles published in 2021 (*n* = 21) not included in figure.

The final conceptual model is presented in Figure 2. A total of 188 studies were included, comprising 100 studies examining a pediatric population, 86 examining an adult population, and 2 examining a population across the lifespan. Studies were published from 1989 to 2021, with a trend towards greater interest in more recent years (see Figure 3). Primary authors were from a total of 34 different countries, most frequently from the United States (*n* = 30), followed by Brazil (*n* = 19), the United Kingdom (*n* = 17), Sweden (*n* = 16), Finland (*n* = 13), and India (*n* = 10).

### 3.1. Relationships between Person Factors

#### 3.1.1. DFA: General Anxiety/Fear

Thirty articles examined the association between DFA and general anxiety/fear (*n* = 11 pediatric, *n* = 19 adult). The majority of both pediatric [43,45,46,47,48,49,50,51,52,53] and adult studies [29,39,54,55,56,57,58,59,60,61,62,63,64,65,66,67,68] reported a relationship between DFA and general anxiety/fear. Only one pediatric study [69] and two adult studies [13,70] found no significant association between general anxiety/fear and DFA.

#### 3.1.2. DFA: Sensory Over-Responsivity

Ten articles reported a relationship between DFA and sensory sensitivities (*n* = 6 pediatric, *n* = 4 adult). From caregiver reports, child differences in sensory processing were found to be significantly associated with DFA [43]; in a study examining dental-anxiety-provoking stimuli, children reported being mildly to severely afraid of multiple sensory-related stimuli during dental care (e.g., tactile, olfactory, gustatory, visual, and vestibular) [71]. In four qualitative studies exploring factors contributing to children’s DFA, the resulting themes included sensory-related challenges triggered by tactile, visual, auditory, olfactory, and/or gustatory stimuli [72,73,74,75]. In a study of adolescents and young adults, sensory-related aspects of previous negative dental experiences (e.g., sight, smell, and sounds of dental treatment) were significantly associated with dental fear [76]. In adults, SOR was also linked to DFA [13,38,77], with two sensory-processing patterns related to over-responsivity (sensory sensitivity and sensation avoiding) significantly associated with high levels of DFA in a sample of female undergraduates [13], and the majority of dentally anxious adults participating in a qualitative interview study reporting that the sight and sound of dental equipment, the smell of the environment, and the vibration of the drill was distressing and increased DFA [38].

#### 3.1.3. General Anxiety/Fear: Sensory Over-Responsivity

The relationship between SOR and general anxiety/fear was examined in eleven studies (*n* = 5 pediatric, *n* = 6 adult). SOR was significantly associated with higher levels of general anxiety/fear in children [43,78,79] as well as adults [13,80,81,82,83]. One study of preschool children found that 43% of those with SOR had a concurrent anxiety disorder, and that symptoms of SOR in preschool significantly predicted general anxiety symptoms at age six [84]. In another study, children with elevated SOR were four times more likely to have clinically relevant internalizing scores, including signs of anxiety [85]. In adults, two sensory processing patterns related to SOR (sensory sensitivity and sensation avoiding) were significantly correlated with increased state and trait anxiety [86].

### 3.2. Relationships between Oral Health Behaviors and Outcomes and Person Factors

#### 3.2.1. Oral Health: DFA

Seventy-nine studies examined the relationship between DFA and oral health (*n* = 41 pediatric, *n* = 38 adult). A significant association was found between high DFA and poor oral health-related quality of life (OHRQoL), a multidimensional measure evaluating oral health status and its related functional and psychosocial impacts [87], in children [88,89,90,91,92] as well as adults [93,94,95,96,97,98,99,100,101,102]. Significant relationships were also reported between high levels of DFA and measures of poor oral health (e.g., caries experience, gingival health, and toothbrushing frequency) in children [3,5,6,7,17,50,51,53,103,104,105,106,107,108,109,110,111,112,113,114,115,116,117,118,119,120,121,122] and adults [4,61,65,66,93,99,102,123,124,125,126,127,128,129,130,131,132,133,134,135,136,137,138,139,140,141,142]. Nine pediatric studies [143,144,145,146,147,148,149,150,151] and three adult studies [56,63,151,152] found no significant associations between DFA and poor oral health or OHRQoL measures.

#### 3.2.2. Oral Health: General Anxiety/Fear

Eight studies (*n* = 3 pediatric, *n* = 5 adult) examined the relationship between general anxiety/fear and oral health. Children with higher general anxiety/fear were significantly more likely to have caries [108] as well as significantly decreased toothbrushing frequency [153]. One study of adults reported that increased general anxiety/fear was significantly associated with worse OHRQoL [94]. The remaining four studies reported mixed results with various outcome measures. General anxiety/fear was significantly associated with: decayed, missing, and/or filled teeth (DMFT) [39,154]; self-perceived dental problems but not oral symptoms in the past 12 months [58]; and recurrent but not overall caries [150]. One study found no association between general anxiety and caries status [152].

#### 3.2.3. Dental Attendance: DFA

The relationship between DFA and dental attendance was examined in 57 articles (*n* = 15 pediatric, *n* = 42 adult). The avoidance of or infrequent dental visits were linked with higher levels of DFA in children and adolescents [3,7,45,50,110,121,148,155,156,157,158,159] as well as adults [4,8,21,39,40,54,57,61,62,63,65,66,101,102,125,126,129,131,133,135,136,138,140,142,160,161,162,163,164,165,166,167,168,169,170,171,172,173]. Sporadic dental attendance as a child, compared to regular attendance, was associated with increased DFA in adulthood [174]. In a qualitative study interviewing dentally anxious adults, all participants expressed that they avoided going to the dentist (e.g., missed appointments, infrequent attendance, and several years of non-attendance), and some reported avoiding care despite the presence of acute pain [38]. Conversely, one study reported a significant relationship between greater child DFA and the likelihood of a dental visit in the past 12 months [175]. Only four studies found no association between DFA and attendance in children [113,119] or adults [134,176].

#### 3.2.4. Dental Attendance: General Anxiety/Fear

The relationship between general anxiety/fear and dental attendance was examined in seven studies (*n* = 7 adult). Four studies reported a significant association between irregular attendance and increased general anxiety/fear [40,56,57,58], whereas three studies found no significant association [39,160,169].

#### 3.2.5. Dental Behavior Management Problems: DFA

Twenty-eight articles explored the relationship between dental behavior management problems (DBMPs) and DFA (*n* = 26 pediatric, *n* = 2 adult). In children, DBMPs were found to be significantly associated with high DFA in both retrospective [41,50,177,178,179,180,181] and prospective studies [9,10,52,114,145,146,157,182,183,184,185,186,187,188,189,190,191]. One study found that over 75% of children with high DFA had a history of DBMP [192], and another reported that dentally anxious children were almost 2.5 times more likely to behave negatively during care [9]. One study found mixed results with a significant association between previous negative behavior and DFA, but no association between current negative behavior and DFA [180]. Only one study reported no association between negative behavior and DFA following adjustment for covariates (e.g., order of dental visits) [193].

The relationship between DFA and DBMPs was only examined in two studies with adult populations. In a sample of university employees, there was a significant correlation between the number of fear behaviors reported (e.g., grabbing the dentist’s hand, refusing treatment, crying) and increased DFA [61]. Additionally, one qualitative study reported that “a few [high DFA adult] participants…admitted being physically aggressive toward the dentist in the past” [38].

#### 3.2.6. Dental Behavior Management Problems: General Anxiety/Fear

Five studies examined the relationship between general anxiety/fear and DBMP (*n* = 4 pediatric, *n* = 1 adult). In children, a history of DBMP was significantly associated with increased general anxiety/fear [9,41,50]. In a sample of dentally anxious adults, those with high trait anxiety were 2.4 times more likely to be difficult to treat (e.g., inability to open the mouth and cooperate with oral examination) [194]. Only one study reported no association between negative behavior during treatment and general anxiety/fear [52].

#### 3.2.7. Dental Behavior Management Problems: Sensory Over-Responsivity

Four studies examined the association between sensory sensitivity and DBMPs (*n* = 3 pediatric, *n* = 1 adult). Children’s negative reactions to auditory, tactile, olfactory, and movement stimuli were significantly associated with a lack of cooperation during dental treatment [37,43]. Negative reactions to touch and noise were also associated with a need for behavior management strategies during previous treatment [37]. Based on survey results, significantly more parents of children with oral SOR reported that it was moderately–extremely difficult for the dentist to clean their child’s teeth, that the child’s uncooperative behaviors increased at the dentist, and that the dentist used restraint often or almost always during prophylaxis, compared to parents of children without oral SOR [195]. In addition, adults that were challenging to treat due to difficulties with behavioral cooperation were significantly more likely to report fearing dental-related smells, sounds, and tactile experiences [194].

#### 3.2.8. Use of Pharmacological Interventions: DFA

Sixteen studies examined the relationship between DFA and the need for pharmacological intervention for dental treatments, including extractions, restorations, prophylaxis, and/or radiography (*n* = 9 pediatric, *n* = 5 adult, *n* = 2 lifespan). Five studies found that DFA was the first or second most commonly reported reason for the use of pharmacological methods to treat children [11,196,197,198,199]; six additional studies reported that increased dental fear in children was significantly associated with a history of treatment under general anesthesia and/or sedation [3,110,111,200,201,202]. In adults, DFA was the second most common reason for referral for treatment under general anesthesia [11], increased DFA was significantly associated with an increased likelihood of referral for sedation [8,203,204,205], and 85% of adults with high DFA reported that they were possibly or definitely interested in receiving future treatment with sedation or GA [161]. DFA was reported to be the most common reason for treatment under general anesthesia throughout the lifespan (3–66 years), irrespective of gender, except for males aged 12–17 years [199].

### 3.3. Relationships between Oral Health Behaviors and Outcomes

#### 3.3.1. Oral Health: Dental Attendance

Eighteen articles examined the relationship between oral health and dental attendance (*n* = 4 pediatric, *n* = 14 adult). In children, irregular dental attendance was associated with DMFT [7,122,206] and poor OHRQoL [207]. In 13 studies of adults, irregular dental attendance was associated with poor oral health (e.g., DMFT and self-report of poor oral health) [8,56,66,96,100,125,135,160,167,169,171,208,209]. One study reported no significant association between dental attendance and change in OHRQoL over a 12-month period in adults [210].

#### 3.3.2. Oral Health: Pharmacological Methods

The association between oral health and the need for pharmacological methods during treatment was examined in 12 articles (*n* = 7 pediatric, *n* = 3 adult, *n* = 2 lifespan). Poor oral health was reported as the first [196,197] and third [11] most common reason for treatment under general anesthesia for children and the second or third most common across the lifespan [11,199]. Children who had undergone treatment under general anesthesia had significantly more decayed, filled, and/or extracted teeth than those who had never undergone general anesthesia [110,202,211]. One study reported that approximately 11% of adult referrals for treatment under general anesthesia were due to an excessive need for treatment [11], and two studies of adults found that those who were treated with sedation had significantly worse oral health [203,204]. Two studies did not support the link between oral health status and the need for care using pharmacological methods [8,212], and one study found that oral health (DMFT score) increased the odds of choosing general anesthesia for restorative treatment, but not to a significant degree [200].

#### 3.3.3. Dental Behavior Management Problems: Pharmacological Methods

The relationship between DBMPs and the use of pharmacological methods was explored in three studies (*n* = 2 pediatric, *n* = 1 lifespan). In one study, extreme non-cooperation was found to be the most common reason for a general anesthesia referral for both children and adults [11]. Uncooperative behaviors and severe management problems contributed to referrals for sedation in one study [213], whereas 63% of children with a history of DBMP reported undergoing treatment with sedation at one or more previous appointments [214].

#### 3.3.4. Dental Attendance: Pharmacological Methods

The relationship between dental attendance and the use of pharmacological methods was examined in four studies (*n* = 1 pediatric, *n* = 3 adult). One study found that children who went to the dentist only when something was wrong were 2.5 times more likely to have undergone treatment using general anesthesia [202]. Adults with irregular attendance patterns were significantly more likely to be referred for treatment using sedation [8,203]. One article reported no significant association between attendance and the use of pharmacological methods in adults [204].

## 4. Discussion

This model provides a more comprehensive view of the relationship between person factors and oral health behaviors and outcomes than previously reported in the literature. For instance, our review found supporting evidence for relationships between DFA, general anxiety/fear, SOR, as well as multiple oral health behaviors and outcomes. The explicit linking of these constructs highlights connections that may be targeted in the development of new interventions to improve oral health behaviors and outcomes as well as the experience of DFA. Because only 3% of the studies examined causal links between constructs, more research is necessary to determine the directionality of these relationships and the precise impacts that these person factors may have on oral health behaviors and outcomes.

Additional relationships exist between the constructs included in our model; however, lines were included in our final model only when links were supported by a minimum of three independent studies (see Figure 1 for the model including relationships below the stated threshold). Fewer than three studies reported relationships between: SOR and pharmacological methods [37,195], general anxiety/fear and pharmacological methods [204], and SOR and irregular attendance [195]. Additionally, relationships between oral health behaviors and outcomes were reported below our minimum threshold, including: DBMP and poor oral health [179,215] and DBMP and irregular attendance [179]. Lastly, although this model focuses on DFA, general anxiety/fear, and SOR, there are other person factors, such as catastrophizing, which may also play a role in dental challenges. Catastrophizing refers to “an exaggerated negative orientation toward stressful or painful situations” [216] (p. 123) and has been associated with poor oral health in one study [88]. Interestingly, research has also reported relationships between catastrophizing and the other person factors reported here—SOR [217,218], general anxiety/fear [219], and DFA [38,73,88,139,155,216,220]. It is important to continue to examine the relationships between these constructs in future research.

Although the relationship between general anxiety/fear, DFA, and oral health behaviors and outcomes has previously been studied, the inclusion of SOR in the understanding and conceptualization of DFA and oral health outcomes and behaviors for neurotypical individuals represents a new addition to the literature. Historically, dental research examining SOR and oral health behaviors and outcomes has focused on clinical populations, including children with autism spectrum disorder, Down syndrome, and attention-deficit/hyperactivity disorder. In these groups, sensory sensitivities have been linked to dental-related challenges in the home and/or clinic environments [42,43,44,221,222,223,224,225,226,227,228,229,230,231,232,233], with the National Institute of Dental and Craniofacial Research [234] suggesting that minimizing sensory stimuli in the dental environment may support the reduction of uncooperative behaviors in children with autism spectrum disorder.

Many of the interventions designed to address DFA in children, such as modeling or distraction techniques, have limited evidence to support their use [15,28,235,236]. Research findings examining distraction and modeling techniques are mixed, with some studies reporting a decrease in DFA, whereas others found either mixed results or no difference (e.g., [237,238,239,240,241,242,243,244,245,246,247,248,249,250,251,252,253,254]). Tell–show–do, one of the most popular techniques utilized by dentists, likewise has little evidence to support its efficacy; however, it is widely accepted by children and parents, and there are no contraindications for its use [28]. In addition, many review articles note the lack of quality and certainty of the evidence of these intervention studies [28,255,256,257]. For individuals with extreme levels of DFA, psychological interventions (e.g., cognitive behavioral therapy, systematic desensitization, exposure therapy) have shown success in reducing DFA and increasing dental attendance [204,238,258,259,260,261,262,263,264,265,266,267]; however, dentists may encounter challenges utilizing and implementing these types of training- and time-intensive intervention techniques [238,268].

Sensory-based methods of stress and anxiety management have been proposed to decrease DFA in dental patients. For example, the “4S principle” aims to reduce four triggers of DFA in the environment: sights (e.g., needles and drills), sounds (e.g., drilling), sensations (e.g., vibrations), and smells (e.g., clinical odors) [269,270]; however, this intervention has not yet been studied. The utilization of other sensory-based interventions during dental care has been investigated, with most reporting preliminary success. For example, aromatherapy was found to have a calming effect for children during dental care and adults in the dental office waiting room [271,272,273,274,275]. Heart rate and physiological anxiety were reduced during dental treatments when using a weighted blanket for deep pressure sensations [276,277,278]. Lastly, a sensory adapted dental environment, designed to decrease noxious stimuli and increase calming stimuli, decreased physiological and behavioral distress in typically developing children and those with disabilities [44,279,280,281,282,283]; this intervention is now included in the American Academy of Pediatric Dentistry’s [284] list of best practices as a potential basic behavior guidance technique for use with dental patients with anxiety or special healthcare needs.

Given the high prevalence of both DFA and SOR in neurotypical individuals, a call for action in both practice and research is appropriate. For example, screening for DFA and SOR using simple pre-visit questionnaires [229] will provide additional information about patient experience, allowing the dentist to then approach treatment with greater knowledge and confidence as well as the option to implement strategies to improve care. Lastly, research is needed to examine the predictive contribution of DFA and SOR to oral health behaviors and outcomes, as well as the rigorous examination of interventions designed to target these constructs in efforts to improve care for those with DFA and/or SOR.

Although this model highlights important factors which should be considered in the discussion surrounding DFA, several limitations should be noted. First, multiple challenges arose when synthesizing the included studies to develop our model, specifically regarding differences in construct naming, definition, and measurement. Multiple studies utilized the same assessment tool but referred to the outcome by different names. For example, the Children’s Fear Survey Schedule—Short Form (CFSS-SF) was stated to measure *general anxiety* in some studies (e.g., [49,50]) but *general fear* in others (e.g., [48]). Due to this challenge, we utilized the broader *general anxiety/fear* term in our model. In regard to construct measurement and definition, DFA was frequently assessed using many different tools (e.g., Children’s Fear Survey Schedule—Dental Subscale, Modified Child Dental Anxiety Scale, Dental Anxiety Question, and Dental Anxiety Survey); however, even in studies utilizing the same tool, there was often variability in the cut-off points for defining the presence/absence of DFA and/or the categorization of high versus low levels of fear (e.g., [94,148]). Second, a PRISMA flowchart was not created because the literature searches that informed the development of this review and model were conducted in an inductive and iterative manner. Lastly, the search strategy for this study included only one database; a systematic review and meta-analysis including multiple databases is a necessary next step to confirm the findings reported here.

## 5. Conclusions

This model contributes to the literature by including other person factors such as general anxiety/fear and sensory over-responsivity to the conversation surrounding DFA and oral health behaviors and outcomes. It is important to consider these factors as they may exacerbate the challenges experienced in the dental clinic, possibly in individuals both with and without comorbid DFA. In addition, knowledge regarding the association between DFA, these additional person factors, and oral health behaviors and outcomes have the potential to inform the development of more targeted interventions to improve care for this population.
